# Expression, maturation and turnover of DrrS, an unusually stable, DosR regulated small RNA in *Mycobacterium tuberculosis*

**DOI:** 10.1371/journal.pone.0174079

**Published:** 2017-03-21

**Authors:** Alexandra Moores, Ana B. Riesco, Stefan Schwenk, Kristine B. Arnvig

**Affiliations:** Institute for Structural and Molecular Biology, University College London, London, United Kingdom; Infectious Disease Research Institute, UNITED STATES

## Abstract

*Mycobacterium tuberculosis* depends on the ability to adjust to stresses encountered in a range of host environments, adjustments that require significant changes in gene expression. Small RNAs (sRNAs) play an important role as post-transcriptional regulators of prokaryotic gene expression, where they are associated with stress responses and, in the case of pathogens, adaptation to the host environment. In spite of this, the understanding of *M*. *tuberculosis* RNA biology remains limited. Here we have used a DosR-associated sRNA as an example to investigate multiple aspects of mycobacterial RNA biology that are likely to apply to other *M*. *tuberculosis* sRNAs and mRNAs. We have found that accumulation of this particular sRNA is slow but robust as cells enter stationary phase. Using reporter gene assays, we find that the sRNA core promoter is activated by DosR, and we have renamed the sRNA DrrS for DosR Regulated sRNA. Moreover, we show that DrrS is transcribed as a longer precursor, DrrS+, which is rapidly processed to the mature and highly stable DrrS. We characterise, for the first time in mycobacteria, an RNA structural determinant involved in this extraordinary stability and we show how the addition of a few nucleotides can lead to acute destabilisation. Finally, we show how this RNA element can enhance expression of a heterologous gene. Thus, the element, as well as its destabilising derivatives may be employed to post-transcriptionally regulate gene expression in mycobacteria in combination with different promoter variants. Moreover, our findings will facilitate further investigations into the severely understudied topic of mycobacterial RNA biology and into the role that regulatory RNA plays in *M*. *tuberculosis* pathogenesis.

## Introduction

In spite of significant endeavours in both drug discovery and vaccine development, tuberculosis (TB), caused by *Mycobacterium tuberculosis*, remains a global threat to human health. Drug treatment is lengthy and complex often with severe side effects, especially for patients with multidrug resistant TB (MDR-TB) and extensively drug resistant TB (XDR-TB). MDR-TB accounted for an estimated 480 000 incidents in 2014, with almost 10% of those being XDR-TB [1]. Moreover, the *Mycobacterium bovis* BCG vaccine does not always offer the desired or even expected protection, and in most cases protection only lasts a decade or two [2]. It is therefore essential that we investigate all aspects of *M*. *tuberculosis* gene regulation in order to understand the host-pathogen interactions important for establishing and maintaining infection. A substantial volume of literature describes stress induced transcriptional changes in the *M*. *tuberculosis* gene expression program [3–6]. Less well-known are the post-transcriptional changes mediated by regulatory RNAs such as riboswitches and small regulatory RNAs (sRNAs).

sRNAs are widely acknowledged as increasingly important players in pathogen gene regulation, where they support and complement the more well-characterised, protein-based gene regulation, in most cases by modulating mRNA stability, reviewed in [7–9]. Many sRNAs are associated with stress responses, adaptation to changing (host) environments and modulation of cell surface components or secreted proteins affecting the host-pathogen interfaces, e.g. [10, 11]. Recently, this has also been shown to be the case in *M*. *tuberculosis* where the missing link between the PhoPR two-component system and the aberrant secretion of TAT-dependent proteins turned out to be the Mcr7 sRNA [12]. However, in spite of several publications describing the identification of novel, putative regulatory RNAs in *M*. *tuberculosis* e.g. [13–18], little is known about their expression, turnover and targets.

The level of any given transcript reflects a balance between synthesis and turnover, and RNA is degraded by a range of endo- and exonucleases with different specificities. While *M*. *tuberculosis*, and its non-pathogenic relative, *Mycobacterium smegmatis*, share some RNases with model organisms such as *Escherichia coli* and *Bacillus subtilis*, there are significant differences in the complement of major RNases associated with mRNA turnover. Thus, mycobacteria contain functional homologues of both RNase E and RNase J, while RNase E is absent from *B*. *subtilis* and RNase J is absent from *E*. *coli* (Table 1), [19–21]. Mycobacterial RNase J (Rv2752c/MSMEG_2685) has dual endo- and exonuclease activities and is capable of endonucleolytic cleavage very close to the 5’ end [20]. Curiously, in spite of the difference in GC content between these organisms (Table 1), the substrate specificity of the mycobacterial RNase E differs only slightly from that of *E*. *coli* RNase E, displaying a preference for A/U-rich sequences [22, 23]. The substrate specificity of mycobacterial RNase J is not known, but it is also believed to be A/U-rich sequences similar to RNase E and to *B*. *subtilis* RNase J1 [20, 24]. The endonucleolytic activity of *E*. *coli* RNase E as well as the exonucleolytic activity of *B*. *subtilis* RNase J are both sensitive to the phosphorylation state of the 5’ nucleotide of their substrates, i.e. they both have a strong preference for mono-phosphorylated over tri-phosphorylated transcripts as substrates due to the presence of specific ‘monophosphate binding pockets’ in these enzymes [25–28]. RNA 5’ monophosphates can be generated either by endonucleolytic cleavage of a transcript or by the removal of a pyrophosphate group from the 5’ nucleotide by the action of the Nudix hydrolase, RppH (RNA pyrophosphohydrolase) [29]. Either way, the monophosphate ‘tags’ the transcript for further, rapid degradation by RNase E or RNase J. So far, there are no reports of RppH homologues in mycobacteria. To fully understand regulation of gene expression in *M*. *tuberculosis*, it is therefore essential to experimentally determine how RNA may be stabilised or degraded in this pathogen.

**Table 1:** Species specific occurrence of nucleases relevant for this study.

Species	RNase E	RNase J	RppH	GC content
*E*. *coli*	yes	no	yes	51%
*B*. *subtilis*	no	yes	yes	44%
*M*. *tuberculosis*	yes	yes	?	66%
*M*. *smegmatis*	yes	yes	?	67%

We are interested in riboregulation associated with stress responses in *M*. *tuberculosis*. One of these is the DosR response, which is induced by a number of infection-associated stresses including hypoxia and NO stress, and it leads to the expression of more than 50 genes involved in adaptation to hypoxic conditions [3, 30]. The DosR regulon is also induced when cells enter stationary phase, but to a lesser extent [13, 31]. DosR is the response regulator of the two-component system DosRS (or DosRT), and it binds cooperatively to multiple DosR binding sites of which the primary (promoter proximal) site overlaps with the -35 region of DosR regulated promoters [32]. The *M*. *tuberculosis* sRNA, MTS1338 (or ncRv11733 according to a more recent annotation [33]) was first identified by RNA sequencing (RNA-seq) and its accumulation in stationary phase is highly dependent on DosR [13].

Here we characterise in detail the expression, processing and turnover of MTS1338 to obtain insights into the RNA biology of this major pathogen. We have investigated the expression pattern of MTS1338 under different growth conditions and by employing reporter gene assays, and we provide evidence that the mature sRNA is generated by 3’ processing of a longer nascent transcript. Moreover, we show that the mature MTS1338 has an unexpectedly long half-life of several hours, and we show that this stability can be perturbed by changing the 5’ structure of MTS1338. Based on our results we propose that the MTS1338 5’ stem-loop makes the sRNA inert to the action of an as yet unidentified mycobacterial RppH homologue, which otherwise initiates RNA turnover by changing the phosphorylation state of the 5’ nucleotide to make it susceptible to the action of RNases E and J. The *M*. *tuberculosis* structural determinants and the pattern of RNA processing identified here are likely to pertain to a plethora of *M*. *tuberculosis* coding as well as non-coding RNAs, thus paving the way for further exploration of *M*. *tuberculosis* RNA biology. Moreover, our findings can be applied to the engineered expression of *M*. *tuberculosis* genes in order to adjust expression levels for synthetic and/or systems biology studies or for vaccine development for an optimized immune response.

## Results

### Expression of a DosR-regulated *M*. *tuberculosis* sRNA

The DosR regulon of *M*. *tuberculosis* has been intensely studied for more than a decade, but its role in persistence and pathogenesis remained inconclusive until recently, when Mehra et al. published evidence for the inability of a *dosR* knockout strain (Δ*dosR*) to cause disease [34].

However, many of the DosR regulon members remain uncharacterised, and among those is the sRNA MTS1338, which was identified by RNA-seq [13]. This sRNA accumulates to high levels in stationary phase and further during chronic mouse infection. In addition, by using a Δ*dosR* strain we demonstrated that the accumulation of MTS1338 was dependent on DosR [13], a key mycobacterial regulator. We have therefore renamed the sRNA ‘DrrS’ for DosR Regulated sRNA.

Preliminary analysis has suggested significant changes in gene expression mediated by DrrS. However, an important issue for any regulator is to ensure that regulation occurs at the right time and place. Therefore, before characterising the DrrS regulon, we wanted to characterise the expression and turnover of the regulator itself, specifically in order to obtain a more comprehensive picture of its role in regulating the expression of other genes, and more generally to gain more insight into mycobacterial RNA biology as a whole.

**Fig 1:**
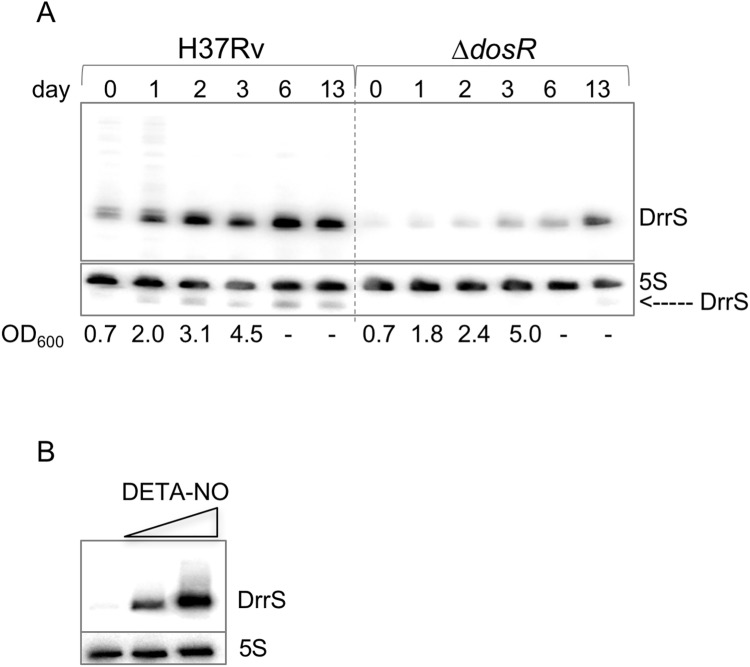
Accumulation of DrrS. A: Comparison of DrrS accumulation between H37Rv and Δ*dosR* over two weeks shown by Northern blotting. Total RNA (5 μg) from the two strains was separated on a single 8% denaturing acrylamide gel and probed for DrrS. The 5S loading control also shows trace amounts of DrrS, particularly in H37Rv, demonstrating that DrrS is slightly smaller than 5S. OD_600_ measurements after day 3 were unreliable due to clumping. B: Induction of DrrS expression with DETA-NO. Cells were grown to mid-log phase and exposed to 0.1 mM and 1.0 mM DETA-NO for two hours, respectively. RNA was analysed by Northern blotting.

First, we investigated the accumulation of DrrS as the cells progressed from exponential growth to late stationary phase (two weeks post log phase). Cultures of *M*. *tuberculosis* were grown to mid-log phase (Day 0, OD_600_ = 0.6), total RNA was harvested on days 0, 1, 2, 3, 6 and 13 and DrrS expression was measured by Northern blotting. Since we had previously observed that the accumulation of DrrS was DosR dependent, we performed the same time course experiment with the Δ*dosR* strain [3]. The results, shown in Fig 1A, demonstrate that in H37Rv DrrS accumulated slowly but to high levels as the cells progress into late stationary phase. As expected, the increase in DrrS levels was significantly slower in the Δ*dosR* strain, confirming that the accumulation of DrrS is highly dependent on DosR. In addition to the main signal, we also observed a series of faint bands corresponding to larger transcripts in wildtype RNA at day 0 and day 1. Visualising the 5S loading control along with DrrS confirmed that DrrS is slightly smaller than 5S, i.e. approximately 110 nucleotides (Fig 1A). Moreover, we also established that DrrS accumulation continued at least three weeks after log-phase (S1 Fig).

### Nitric oxide dependent expression of DrrS

As the accumulation of DrrS in stationary phase is DosR dependent, we investigated DrrS expression and accumulation under a condition known to induce DosR, namely Nitric Oxide (NO) [35]. Cultures of *M*. *tuberculosis* were grown in standard 7H9 medium to mid-log phase (OD_600_ = 0.6) and DETA-NO was added at final concentration of 0.1 and 1.0 mM, respectively. The cultures were harvested after two hours of NO stress and total RNA was analysed by Northern blotting. As seen in Fig 1B, the addition of DETA-NO led to a dose-dependent induction of DrrS, which was much more rapid than the slow accumulation seen in stationary phase.

Other stressors such as hydrogen peroxide (oxidative stress), Mitomycin C (DNA damage) and low pH did not result in DrrS induction (not shown), implying that the NO response was highly specific, and corroborating the link to DosR.

**Fig 2:**
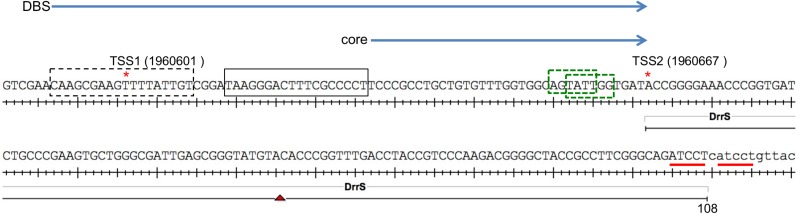
DrrS and its promoter region . Transcription start sites (TSS) are indicated with red asterisks, putative -10 boxes for TSS2 are shown in green; previously identified DosR binding site (DBS) is shown as a black box, while a putative DBS is shown as a black dashed box. Regions cloned as *lacZ* fusions are shown as blue arrows above the promoter sequence. Red lines indicate ATCCT repeats in 3’ region (see text).

To further address the role of DosR, we investigated the *M*. *tuberculosis* DrrS promoter region in more detail. DrrS has two transcription start sites (TSS): a weak one at T1960601 (here referred to as TSS1 expressed from P1) and a strong one at A1960667, (referred to as TSS2, expressed from P2, Fig 2) [13]. A single DosR binding site (DBS), [36] was identified 34 basepairs upstream of TSS2, i.e. overlapping with the -35 region [13], which makes this promoter similar to class II CRP dependent promoters from *E*. *coli* [37]. The distance between the TSS and the DBS is in good agreement with the published canonical distance between DosR associated TSSs and their proximal DBS, suggesting that the DosR dependent transcript is expressed from P2, although a second, distal DBS seems to be missing. As DosR reportedly acts on SigA dependent promoters [38], we identified two AT-rich hexamers (TATTGG and AGTATT), which both deviate slightly from the SigA -10 consensus sequences identified by Cortes (TANNNT) [39] and Shell (A/T ANNNT) [40], upstream of TSS2 (Fig 2). Only one of these (AGTATT) appeared to have the correct spacing to the TSS, suggesting that this is the more likely candidate. In support of this notion we found that several other DosR regulated genes have a similar -10 region (Table 2). As several DosR regulated genes appear to have SigC recognition motifs in addition to SigA motifs [41–43], we scrutinised the region for alternative sigma factor binding sites, but did not find any obvious matches to known consensus sequences [44]. It has been proposed that a minimum of two closely spaced DBS is required for DosR-activated expression [32]. We therefore interrogated the region immediately upstream of the known DBS and identified a sequence that had weak homology to the consensus sequence of both primary and secondary DBS (Fig 2) [32]. Hence, we speculated that this region could possibly serve as a weak or cryptic, secondary binding site capable of supporting the promoter proximal binding site.

**Table 2:** Sequences of -10 regions in selected DosR regulated genes.

Gene	Transcription Start Site (TSS)	-10 region
DrrS	1960667	gccAGTATTggtgat**a**
Rv0079	88166	gccAGACAAgctttgg**g**
Rv0574c	668453	ccgATCGTAtgacgt**g**
Rv1733c	1960476	gcgATGATCccac**a**
Rv1737c (*narK2*)	1965427	gccAGGGTTagcgc**a**
Rv2032	2279105	ttcAGAAAGatcggg**g**
Rv2629	2955519	cacAACGATcgaag**g**

Transcription start sites (TSS) taken from [39]. Sequences in upper case shows putative -10 boxes, bold letter last in each sequence corresponds to the mapped TSS. A shared GCCAG motif in the promoters of DrrS, Rv0079 and Rv1737c has been underlined.

### Reporter gene analysis of DrrS expression

**Fig 3:**
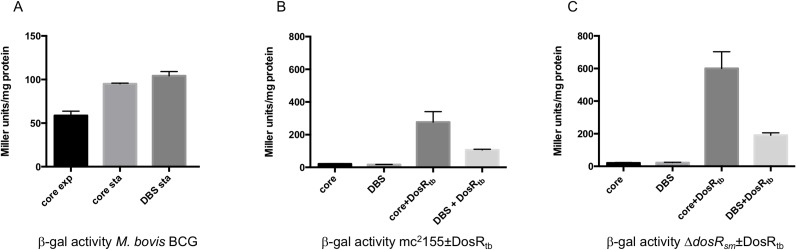
DrrS promoter activity. A: ß-galactosidase assays of DrrS promoter-reporter fusions containing either core or ‘full-length’ promoter, including DBS (Fig 2) were transformed into *M*. *bovis* BCG. Stationary phase cultures (Sta) were grown one week past OD_600_ = 1.0 before harvest and ß-galactosidase assays performed on cell extracts. Results represent mean ± standard deviation of three biological replicates normalised to total protein and values for empty vector subtracted; p<0.05. B: ß-galactosidase assays of DrrS promoter-reporter fusions expressed in *M*. *smegmatis* mc^2^155 or C: Δ*dosR*_*sm*_ [45] with and without *dosR*_*tb*_. The assays were performed on exponential phase cultures and the Results represent mean ± standard deviation of three biological replicates normalised to total protein and values for empty vector subtracted; p<0.05.

To establish the contribution of the DBS to the DosR-dependent expression of DrrS, we made genomically integrating promoter-*lacZ* reporter gene fusions extending upstream from TSS2. A ‘minimal’ construct included the core promoter only, i.e. excluding the DBS, 35 basepairs upstream of TSS2 (‘core’, Fig 2). The second ‘full-length’ construct included core promoter and upstream region with the known DBS as well as the hypothetical additional DBS, in total 75 basepairs upstream of TSS2 (‘DBS’, Fig 2). The constructs were transformed into *Mycobacterium bovis* BCG, which encodes DrrS, RNA polymerase and DosRS proteins identical to those of *M*. *tuberculosis*. ß-galactosidase activity was measured one week after OD_600_ reached 1.0, where DrrS levels are high similar to *M*. *tuberculosis* (data not shown). For comparison, we also measured the ß-galactosidase activity of the core promoter construct in exponential phase and in each case the values for the empty vector control have been subtracted. The results showed that the activity of the minimal promoter construct increased 1.6-fold between exponential and stationary phase. In stationary phase, the full-length promoter construct had the same activity as the core construct, which implies that the upstream DBS-containing region did not confer any further activation (Fig 3A). This suggests that DosR-dependent accumulation of DrrS is not dependent on the canonical DosR-mediated mechanism of activation of transcription. To shed more light on these results and on the role of DosR in DrrS transcription, we cloned the *M*. *tuberculosis dosR* gene (*dosR*_*tb*_) into the same integrating vector as the reporters, but divergently transcribed from a strong, heterologous promoter that is active in exponential phase, where native *dosR* expression is minimal. The heterologous over-expression implies that the expressed DosR_tb_ will be present in larger amounts in the cell than under native conditions, but mostly in its un-phosphorylated state, which has reduced affinity for DNA [41]. The constructs were transformed into *Mycobacterium smegmatis*, which is widely acknowledged as a tractable surrogate host for *M*. *tuberculosis* gene expression. As background, we chose two strains; the wildtype strain mc^2^155 and a derivative, Δ*dosR*_*sm*_, in which the *M*. *smegmatis dosR* gene had been deleted [45] (gift from Huw Williams). The reporter strains were grown to mid-log phase and cell extracts were assayed for ß-galactosidase activity with values for empty vector control subtracted. The results indicated that in the wildtype mc^2^155 background, in the absence of *dosR*_*tb*_ both the minimal and the full-length promoter constructs had very low and very similar promoter activities, i.e. 21 and 17 Miller units/mg protein, respectively (Fig 3B). This result shows that, during exponential growth the activity of the DrrS core promoter is at least two-fold lower in *M*. *smegmatis* than in *M*. *bovis* BCG. When the constitutively over-expressed *dosR*_*tb*_ was included in the vector, both minimal and full length promoter activities were dramatically and significantly increased to 277 and 92 Miller units/mg protein for core and DBS constructs, respectively (Fig 3B). In other words, the minimal core promoter had higher activity than the full-length promoter including the upstream DBS region. Using the Δ*dosR*_*sm*_ strain as background for the same reporter constructs resulted in a similar pattern; i.e. the activities of both minimal and full length promoters core and DBS were low, in this case between 21 and 23 Miller units/mg protein. The over-expression of *dosR*_*tb*_ led to the activation of both promoters, and similarly to the wildtype background, the full-length promoter was activated significantly less than the core promoter (Fig 3C). In this case, the absolute ß-galactosidase activities of the reporters were approximately two-fold higher than the identical constructs in the wildtype background, suggesting that DosR_tb_ activation of the DrrS promoter is higher in the absence of the native DosR_sm_. It should be noted that DosR_tb_ also activated the empty vector control (to 43±7.4 and 50±4.6 Miller units/mg protein for mc^2^155 and Δ*dosR*_*sm*_, respectively), although these values were subtracted from the final, normalised results. All the normalised values including fold increase for each construct have been collated in Table 3.

**Table 3:** β-gal activity of DrrS promoter constructs assayed in *M*. *smegmatis*.

strain/reporter ± DosR_tb_	ß-gal activity	fold increase*
mc^2^155/core	21±1.45	-
mc^2^155/DBS	16.8±0.71	-
mc^2^155/core + DosR_tb_	277±64	13
mc^2^155/DBS + DosR_tb_	91.6±6.2	5
Δ*dosR*_*sm*_ /core	21±2.4	-
Δ*dosR*_*sm*_ /DBS	23±2.2	-
Δ*dosR*_*sm*_ /core + DosR_tb_	601±102	29
Δ*dosR*_*sm*_ /DBS + DosR_tb_	191±15	8

* Increase in activity conferred by expression of DosR_tb_ from same vector as the reporter

### Mapping of the DrrS 3’ end

As scrutiny of the 3’ region of DrrS did not reveal an obvious canonical intrinsic terminator structure or termination sites, we performed 3’ RACE on DrrS. Total RNA from *M*. *tuberculosis* was poly-A tailed and 3’ RACE carried out as previously described [14] using a primer that extended from U40 relative to TSS2 (genome position T1960706). The results showed that in 72% (23 of 32) of the sequenced clones, the 3’ end of DrrS was mapped to U108 relative to TSS2 (genome position T1960774), i.e. the 2^nd^ T (U) residue within the repeat sequence ATCC**T**catcct, where lower case letters refer to the DNA sequence downstream of the DrrS 3’ end (see also Fig 2). The resulting sRNA, when expressed from P1 would therefore be 174 nucleotides long, while the one expressed from the P2 would be 108 nucleotides long, the latter in accordance with the size observed by Northern blotting. The majority of the remaining RACE clones (i.e. 8 out of 9; 25% of total) had one additional cytosine residue at the 3’ end (ATCCT**C**atcct), thereby giving rise to a subpopulation of DrrS transcripts that were 109 nucleotides in length.

### DrrS is processed from a longer transcript

**Fig 4:**
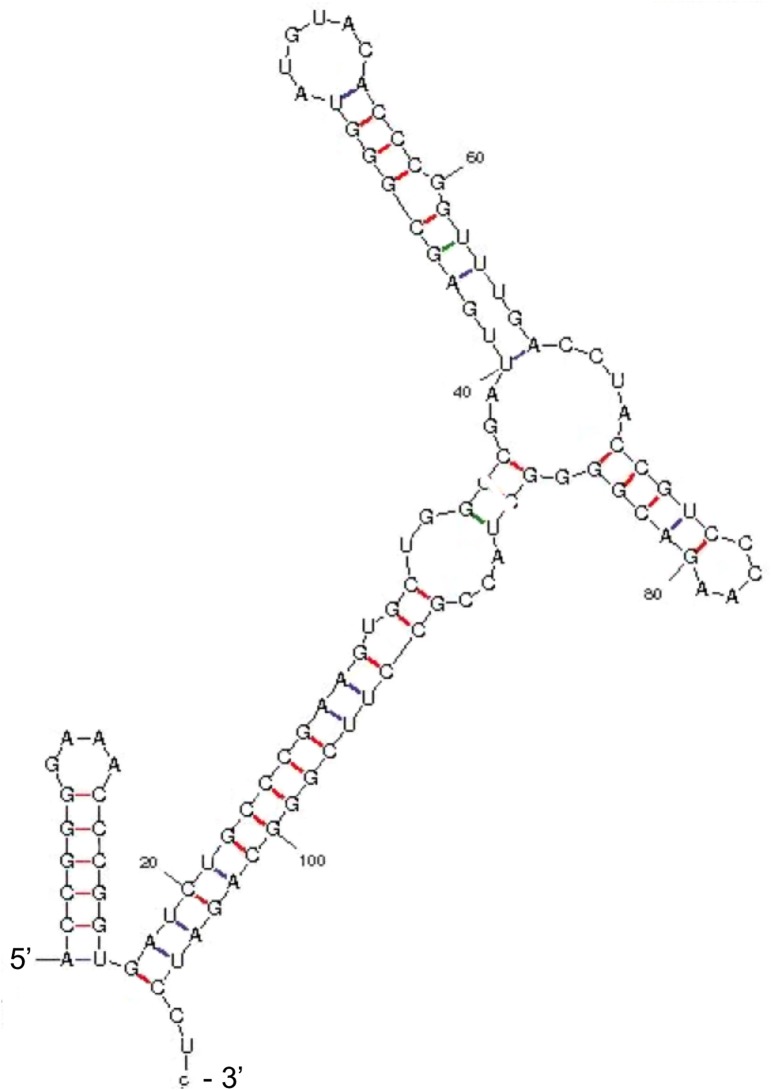
Predicted structure of the 109 nucleotide DrrS. The 109 nucleotide DrrS sequence from TSS2 to the mapped CU(C) 3’ end was used to predict the structure in *mfold*.

**Fig 5:**
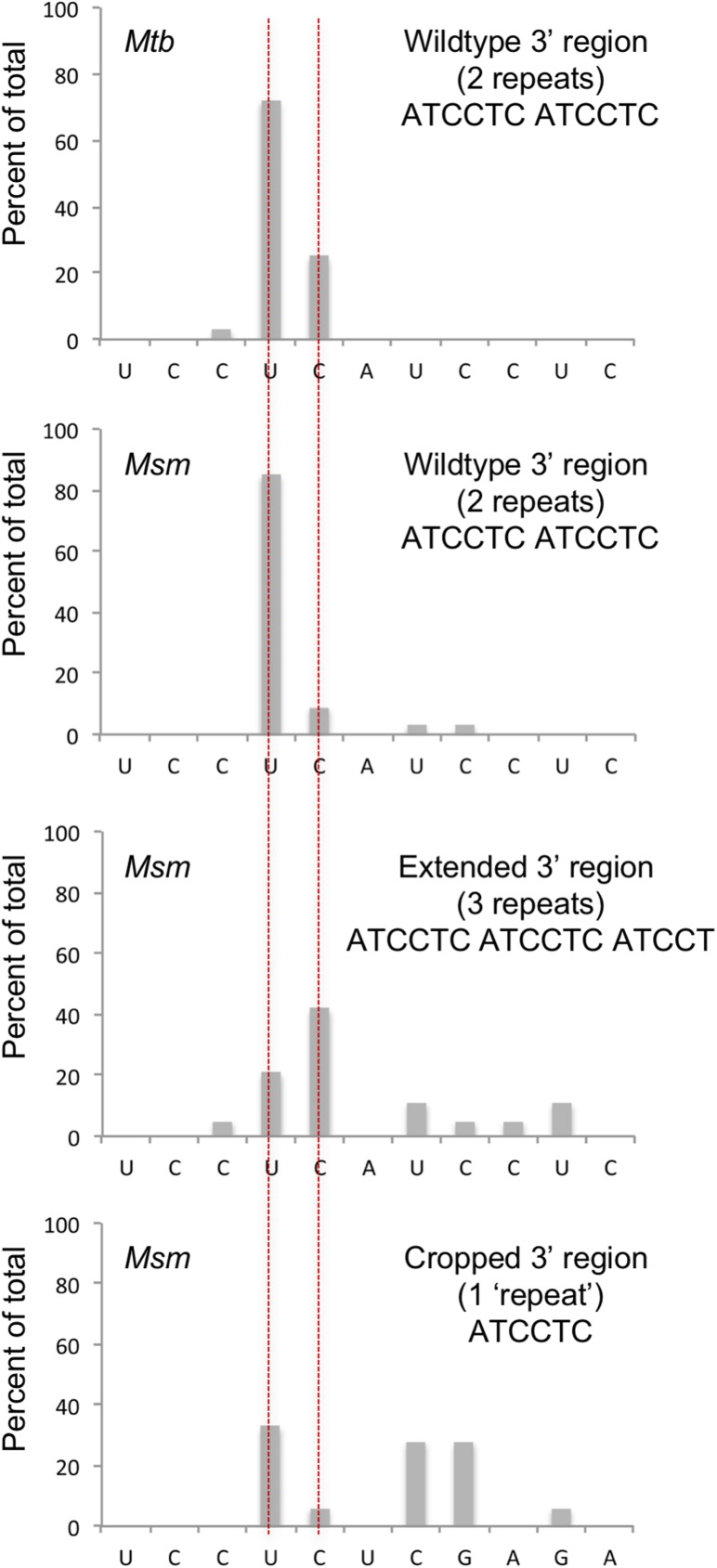
Mapping of 3’ termini of DrrS variants. The panels illustrate how the DrrS 3’ termini vary as the 3’ region is modified. The top panel shows the 3’ RACE results of wildtype DrrS sequence with two ATCCTC repeats, which in *M*. *tuberculosis* leads to the majority of transcripts ending in U or C within the first repeat (indicated with red, vertical lines). Panels 2–4 show the results of DrrS variants expressed in *M*. *smegmatis*; panel 2 represents 3’ RACE results of DrrS cloned with two ATCCTC repeats before the SynB terminator, similar to wildtype DrrS; panel 3 shows the results when an extra repeat has been inserted, and panel 4 the results when only one repeat is included.

The structure of the 109-nucleotide DrrS was predicted using *mfold* [46]. According to this prediction the structure of DrrS begins with a short, 5-basepair, GC-rich stem-loop and finishes with a long imperfect stem and a short CU (108 nucleotide) and CUC (109 nucleotide) tail at its 3’ end (Fig 4). The complete lack of a 3’ poly-U tract makes this structure incompatible with a function as intrinsic terminator, even in *M*. *tuberculosis* [47]. In order to shed more light on DrrS 3’ end formation, we created recombinant DrrS variants in which the template DNA had modified 3’ regions. These variants were expressed from the *rrnB* promoter of *M*. *smegmatis* [48] and included the native DrrS TSS. At the 3’ end they contained either one, two or three of the ATCCT repeats identified in the 3’ region of DrrS (ATCCT_1_, ATCCT_2_, ATCCT_3_) followed by a short XhoI linker and the strong synthetic SynB terminator [47], to ensure efficient termination (methods for details). The DrrS 3’ variants were expressed in the fast-growing and more tractable *M*. *smegmati*s, which harbours the same complement of RNases as *M*. *tuberculosis*, and which does not encode a DrrS homologue. Total RNA was isolated from each strain and the 3’ ends mapped using RACE as described above. The results are shown schematically in Fig 5. The top panel illustrates how the native DrrS 3’ termini fall mainly on U108 and to a lesser extent C109 in *M*. *tuberculosis*. The second panel shows that the heterologous expression of DrrS from the template with two ATCCT repeats (i.e. similar to wildtype DrrS) in *M*. *smegmatis*, mirrors the DrrS 3’ end formation in *M*. *tuberculosis*. Thus, in *M*. *tuberculosis* 97% of 3’ ends fall on U108 or C109 compared to 94% in *M*. *smegmatis*. However, when a third ATCCT repeat was added to the template DNA, we observed a shift in the RNA termini downstream of C109 (Fig 5, panel 3). Similarly, when we removed a repeat, in effect changing the DNA sequence downstream of the native 3’ end, we also observed several transcripts with 3’ additions. The collated results are shown in S1 Table, and together they suggest that the DrrS 3’ CU(C) tail is not generated by termination of transcription but rather by processing of a longer transcript, which we will refer to as DrrS+.

### Mapping of DrrS+ 3’ termini

**Fig 6:**
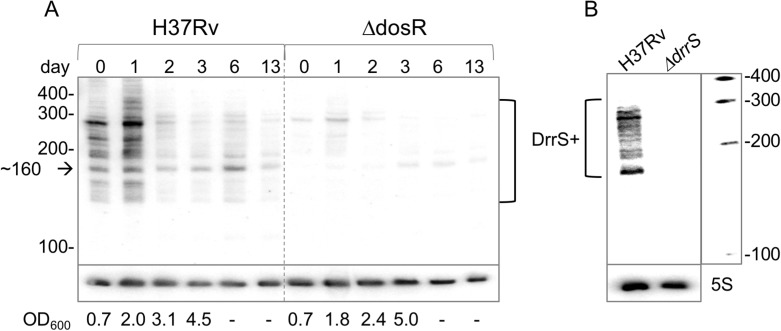
Growth phase dependent accumulation of DrrS+. A: Time course of expression from log- to stationary phase (as in Fig 1), but with more RNA and probed with the DrrS+ probe complementary to RNA downstream of position C109. Arrow indicates prominent transcript around 160 nucleotides described in the text. B: total RNA from *M*. *tuberculosis* H37Rv and *M*. *tuberculosis* Δ*drrS* also probed for DrrS+ showing a range of transcripts similar to A in H37Rv but no detectable transcripts in Δ*drrS*. Marker: Century marker (Ambion).

As the DrrS time course experiment (Fig 1) had indicated the presence of larger DrrS+ transcripts during early stationary phase but not at later time points, we investigated the accumulation of DrrS+ from log phase into stationary phase in H37Rv and Δ*dosR*, similar to what had been done for DrrS. RNA from the same time course was probed, but this time with a DrrS+ probe complementary to a region downstream of C109 to specifically identify DrrS+. The results demonstrate that a series of transcripts between ~160 and ~400 nucleotides were recognised by the DrrS+ probe (Fig 6A) with particularly strong signals at day 0 (log-phase) and day 1 (early stationary phase). These time points were dominated by a signal just below 300 nucleotides, while further into stationary phase a signal of ~160 nucleotides dominated (Fig 6A). A similar pattern was observed in the Δ*dosR* strain, but again with much weaker intensity, and the shift from the larger (~300 nucleotides) to the smaller (~160 nucleotides) transcript is more obvious in this strain, possibly due to the reduced background signals. To ensure that this signal was in fact associated with DrrS/DrrS+ we probed RNA from a Δ*drrS* strain (harvested at OD_600_ = 1.5), from which we observed no signal (Fig 6B), verifying that the transcripts were indeed encoded by the DrrS gene. The presence of several bands up to ~400 nucleotides suggests that either termination of transcription is imprecise or the nascent DrrS+ is >400 nucleotides in length and all of the observed signals arise from processing, or both.

In order to further elucidate DrrS 3’ end generation we performed additional 3’ RACE with primers that extended from C119 and C135 of DrrS+, respectively. The results, shown in S2 Table indicate the presence of several different 3’ termini, which is also reflected in the multiple signals seen on the Northern blots in Fig 6. More specifically, the results also indicate that some 3’ positions were represented by more than one clone (C125, C135, C140, C162, C164), while others were represented only once. C164 was represented by 5 of the 36 clones (14%) and is likely to correspond to the prominent signal seen around 160 nucleotides on the Northern blot (indicated by arrow in Fig 6A), and similar in size to what has been reported recently by Wang et al. [49]. The prominent signal just below 300 nucleotides could correspond to the 3’ termini mapped to 271 and 273. It is worth noting that the ten most stable of the predicted folds of the 273-nucleotide long DrrS+ all include a 5’ structure that is identical to the mature 108 nucleotide DrrS, suggesting that this stem-loop structure is likely to form regardless of the heterogeneity of the 3’ tail (Fig 4).

**Fig 7:**
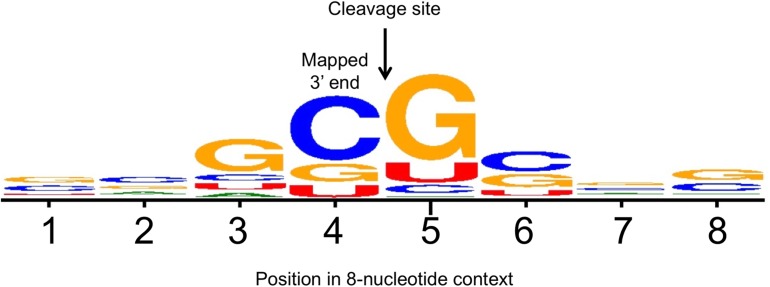
Alignment of DrrS 3’ termini in WebLogo. All the 3’ termini obtained with extended 3’ RACE (i.e. beyond C109) were aligned in WebLogo, which resulted in a GC|G consensus cleavage site.

More than half of all the mapped 3’ termini (19 of 36) fell between a G and a C residue, and 12 of these (33%) were within the sequence GC|GC. This led us to align all of the mapped 3’ termini from C122 to U297 in the context of four nucleotides on either side using WebLogo [50]. The resulting consensus, shown in Fig 7, strongly suggest sequence specific cleavage between C and G within the sequence GC|G with a striking underrepresentation of A, and to some extent U residues in the 8-nucleotide context shown, thereby making it unlikely that these termini are generated by RNase E or RNase J, which have a preference for U- and A/U-rich substrates, respectively. The DrrS+ sequence was interrogated for putative ORFs and we found two; a small one covering positions A50 to U142 and a large one covering positions G15 to A368, relative to TSS2 of DrrS. The latter has been annotated as the hypothetical MT1775 in CDC1551 [51]. Both were tested for translation by fusing these in frame to a *lacZ* reporter, but neither resulted in expression of the reporter (not shown)

### Stability of DrrS

**Fig 8:**
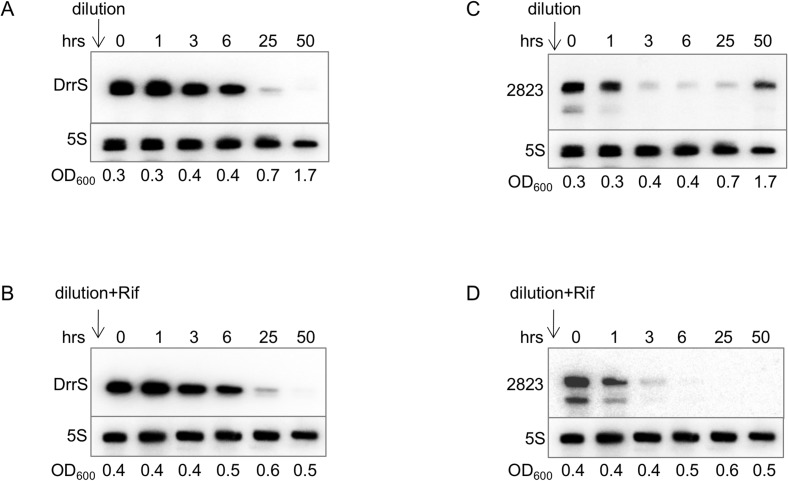
Turnover of DrrS in *M*. *tuberculosis*. A stationary phase culture of *M*. *tuberculosis* OD_600_ ~6 was diluted into fresh, pre-warmed medium to OD_600_ = 0.4 either without rifampicin (panel A and C) or with 200 μg/ml rifampicin (panel B and D) and samples removed for RNA extraction and Northern blotting at the indicated time points. A and B: Northerns probed for DrrS; C and D: Northerns re-probed for MTS2823.

Since the DrrS promoter was shown to be relatively weak and therefore activation of transcription alone was unlikely to account for the significant accumulation of DrrS we observe in stationary phase (Fig 1), and since the amount of any transcript reflects the balance between its synthesis and its turnover, we decided to investigate the stability of DrrS. We made use of the fact that DrrS is expressed at very low levels during exponential growth, i.e. on average < 1 copy per 10 cells [7, 13]. By diluting a stationary phase culture of *M*. *tuberculosis*, where DrrS is present in high amounts into fresh medium to an OD_600_ ~ 0.4, where DrrS is normally present in low amounts, we should only detect DrrS transcripts that were synthesised before the dilution, as DrrS is expressed at very low levels at this stage of growth. For comparison, the dilution was done both in the presence and absence of 200 μg/ml rifampicin. Samples for RNA extraction were removed at times 0, 1, 3, 6, 25 and 50 hours after dilution, and DrrS levels were analysed by Northern blotting. The results shown in Fig 8 demonstrate that both in the presence and absence of rifampicin, DrrS disappears very slowly over time with a significant amount remaining 6 hours after dilution, suggesting that DrrS is extremely stable under these conditions. The fact that the addition of rifampicin had little effect on the disappearance of the DrrS signal, suggests that *de novo* RNA synthesis did not significantly influence the levels of DrrS during exponential growth. To assess if DrrS turnover was different during stationary phase, where DrrS is more abundant, we added rifampicin to a stationary phase culture and sampled RNA over 50 hours. Northern blotting revealed that the stability of DrrS was also considerable in stationary phase (S2 Fig). Finally, we also tested if DosR had an effect on stationary phase stability of DrrS by performing the stability experiment in the Δ*dosR* background. The result, shown in S2 Fig indicate that the stability of DrrS is largely the same in the two strains, suggesting that DosR does not alter the stability to DrrS.

To establish if this level of stability was a general feature of *M*. *tuberculosis* sRNAs, we re-probed the membranes for the MTS2823 sRNA, which is the most abundant non-ribosomal sRNA in stationary phase cells [13]. The result, shown in Fig 8C and 8D, indicates that the MTS2823 signal is significantly reduced after three hours particularly in the presence of rifampicin, suggesting that MTS2823 has a significantly shorter half-life than DrrS during exponential growth. However, both sRNAs still have a substantially longer half-life than that of the most stable *M*. *tuberculosis* mRNA (Rv2886c), which is 18.5 minutes [52]; in the same study the average mRNA half-life was found to be less than 10 minutes.

In summary, our results demonstrate that DrrS is unusually stable and keeping in mind that transcriptional activation of DrrS expression was limited, we conclude that this stability plays a major role in the accumulation of DrrS in stationary phase.

### Structural determinants of DrrS stability

Since we had established that DrrS turnover is slow, we decided to investigate in more detail which RNA motifs might influence the stability of DrrS, and by extension mycobacterial sRNAs and mRNAs in general.

The predicted structure of DrrS, according to both *mfold* [46] and *RNAfold* [53] includes a 5’ terminal stem-loop in which A1 (TSS2) is the first nucleotide of the stem, (Fig 4). 5’ stem-loop structures have long been known to stabilise transcripts in *E*. *coli* [54], and it has been shown in *E*. *coli* and in *B*. *subtilis* that this type of structure is refractory to the action of RppH, which converts 5’ tri-phosphates to 5’ mono-phosphates. Consequently, these transcripts are also refractory to the action of RNases E and J, which are both sensitive to the 5’ phosphorylation state of their substrates [26–28, 55]. Therefore, the presence of unpaired nucleotides 5’ of a stem leads to destabilisation of the RNA by initiating a sequence of nuclease attacks, first by RppH and next by RNases E or J, depending on the species [29, 56].

We hypothesised that the presence of a structure known to impede RppH action in other species, would lead to increased DrrS stability even though no RppH homologue has been identified in mycobacteria. This hypothesis was tested *in vivo* by modifying the 5’ structure of DrrS; if an RppH homologue were involved, the addition of unpaired nucleotides 5’ of the existing TSS should lead to an increased conversion of the 5’ tri-phosphate to mono-phosphate followed by a dramatic destabilisation of the RNA. We created a series of DrrS variants with one to four unpaired nucleotides added 5’ of the native TSS to investigate if this had an effect on DrrS stability. To maintain the same initiating nucleotide as in the wildtype DrrS, the added nucleotides were all adenosine residues (A_1_DrrS to A_4_DrrS). The predicted fold of the variants was tested in *mfold* and they all retained the same basic structure. All variants were expressed from a heterologous, constitutive promoter and contained two ATCCT 3’ repeats similar to wildtype DrrS followed by a six-nucleotide linker and the strong SynB terminator. As with the 3’ variants above, all 5’ variants were expressed in *M*. *smegmatis*, which has the same complement of RNases as *M*. *tuberculosis* [20]. Northern blotting performed on RNA isolated from *M*. *smegmatis* expressing the recombinant, wildtype DrrS and wildtype DrrS expressed in *M*. *tuberculosis* verified that the two sRNAs were identical in size (not shown).

**Fig 9:**
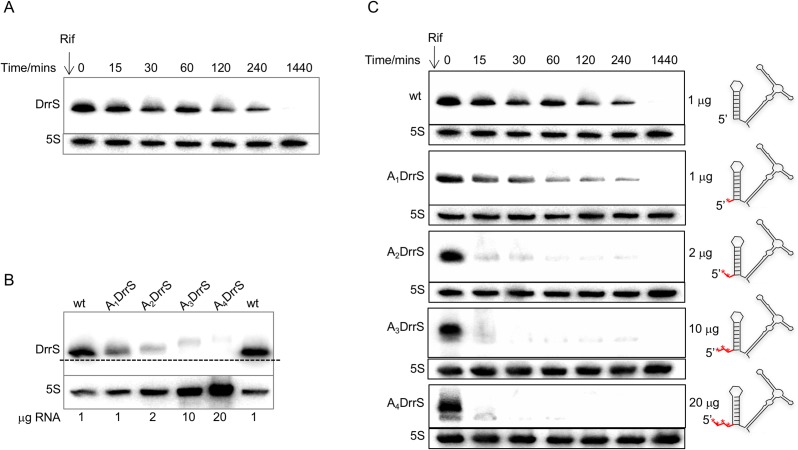
Turnover of DrrS and its 5’ derivatives in *M*. *smegmatis*. A: Northern blot of total RNA from *M*. *smegmatis* expressing DrrS from a heterologous promoter. Rifampicin (200 μg/ml rifampicin) was added at time zero and RNA was isolated at the indicated time point and probed for DrrS as previously described. B: Northern blot of 1–20 μg of total RNA from *M*. *smegmatis* expressing wildtype and 5’ variants of DrrS. RNA from the strain expressing wildtype DrrS was loaded in the first and last wells to ensure proper alignment. C: Turnover of DrrS 5’ variants. Each DrrS 5’ variant was expressed in *M*. *smegmatis*, cultures were grown to OD_600_ = 0.6 and rifampicin added to a final concentration of 200 μg/ml. Samples were withdrawn at the indicated time points and subjected to Northern blotting. As the relative amount of DrrS varied significantly between the wildtype and the derivatives (see panel B), different amounts of total RNA were loaded on the gels, i.e. 1 μg each wildtype and A_1_DrrS; 2 μg of A_2_DrrS; 10 μg A_3_DrrS and 20 μg A_4_DrrS. Cartoon on the right illustrate the 5’ additions (red asterisks) to the DrrS structure.

Initially we tested the stability of DrrS with wildtype TSS in *M*. *smegmatis* by Northern blotting (Fig 9A). The result demonstrates that DrrS remains highly stable when expressed from a heterologous, strong, constitutive promoter in exponential phase in *M*. *smegmatis*. Secondly, we wanted to ensure that the DrrS TSS was shifted according to our predictions and as a consequence that the derivatives increased in size. Hence, in order to compare the sizes of the DrrS wildtype to its derivatives A_1_DrrS through to A_4_DrrS we analysed RNA from all strains by Northern blotting. Initial experiments indicated that the amounts of DrrS variants in the cells decreased significantly as more 5’ A-residues were added, to the extent where we could not detect A_3_DrrS and A_4_DrrS with comparable exposure settings as wildtype and A_1_DrrS (not shown). In order to compensate for this difference in DrrS levels, we loaded increasing amounts of total RNA for the Northern blot such that we probed 20 times more RNA from A_4_DrrS than from wildtype. The results shown in Fig 9B demonstrate that the sizes of the derivatives do increase in a stepwise manner, suggesting that each transcript does indeed have one more nucleotide added 5’ of the previous TSS. Moreover, it is clear that in spite of loading 20 fold more total RNA from the strain expressing the A_4_DrrS 5’ variant there is still significantly more of wildtype DrrS. In essence, the steady-state levels of DrrS variants show an inverse correlation with the number of unpaired 5’ nucleotides. Since both promoter and the initiating nucleotide were identical, this already suggested a faster turnover of DrrS as more unpaired 5’ nucleotides were added.

To explore this phenomenon further, we investigated the kinetics of the turnover of each 5’ derivative after the addition of rifampicin. As before we adjusted the amount of total RNA in the Northern blots so that the time zero samples of each derivative resulted in more comparable signals. The results, shown in Fig 9C demonstrate that the addition of a single 5’ nucleotide has a limited effect on the turnover of DrrS, while the addition of two or more has a profound effect on the turnover of DrrS.

### The phosphorylation state of the 5’ nucleotide is altered *in vivo*

**Fig 10:**
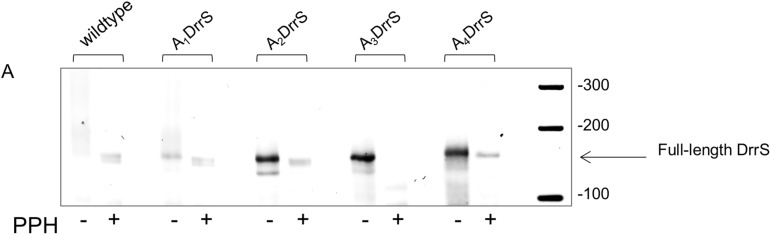
5’ RACE. PCR products from wildtype and DrrS 5’ variants separated on 3% agarose. Total RNA was treated with RNA pyrophosphohydrolase (PPH) before linker ligation, cDNA synthesis and PCR amplification.

Our analysis so far could not discriminate between the following two mechanisms; i) the full-length DrrS variants underwent initial tri- to mono-phosphate conversion by an unknown RppH homologue followed by RNase E or RNase J cleavage; ii) RNase J performed an initial endonucleolytic cleavage very close to the DrrS 5’ end immediately upstream of the stem-loop. If an RppH homologue were involved (i) we would expect a significant proportion of the full-length DrrS variants to be mono-phosphorylated, in particular with increasing numbers of 5’ A-residues. If there were no RppH homologue involved (ii), we would expect all full-length DrrS to be tri-phosphorylated, while mono-phosphorylated DrrS variants would necessarily lack one or more 5’ nucleotides. To ascertain which of these mechanisms apply, we performed RLM-RACE [57] on all DrrS variants. This method exploits the fact that an RNA linker can only be ligated to a 5’ monophosphate using T4 RNA ligase; hence, if a transcript is tri-phosphorylated the linker can only be added after *in vitro* treatment with pyrophosphohydrolase (PPH). The subsequent PCR amplification requires the linker sequence and is therefore dependent on ligation. The resulting PCRs of all the derivatives are shown in Fig 10, which indicates that there is no specific PCR product for wildtype DrrS without prior pyrophosphohydrolase (PPH) treatment, suggesting a very low frequency of full-length mono-phosphorylated 5’ nucleotides; this is in agreement with previous findings [13]. However, it can be seen that the relative amount of PPH independent full-length DrrS increases with increasing 5’ additions, suggesting an increased fraction of full-length mono-phosphorylated transcripts *in vivo*. These results indicate that the 5’ phosphorylation state of the DrrS variants was altered by an RppH homologue *in vivo*, followed by further cleavage of the mono-phosphorylated transcripts. The 5’ RACE results also rules out that the TSS was inadvertently shifted back towards the native TSS, as this would have meant that the shorter variants would be tri-phosphorylated.

We conclude that the absence of unpaired nucleotides at the 5’ end of the wildtype DrrS structure renders the RNA inert to the action of one or more nucleases, and that the addition of unpaired A-residues to the 5’ end destabilises the transcript. A BLAST search with full-length *E*. *coli* and *B*. *subtilis* RppH against *M*. *smegmatis* returned several possible NUDIX hydrolases, but the residues involved in RppH function, according to Foley et al. [58], suggested a closer functional homology to the *E*. *coli* than to the *B*. *subtilis* protein. S3 Fig shows a phylogenetic tree with *E*. *coli* RppH and the *M*. *smegmatis* proteins, which suggests that the closest relatives of the *E*. *coli* RppH are MSMEG_6927 and MSMEG_2390 (homologues of Rv3908/MutT4 and Rv2985/MutT1, respectively).

### The DrrS stem-loop increases expression of reporter construct

In order to establish if the DrrS 5’ stem-loop could lead to increased stability of other transcripts, we tested the expression of the DrrS full-length promoter with and without the stem-loop in our *lacZ*-reporter assay. The stem-loop was added immediately downstream of the TSS such that this was the only difference between the constructs. The expression was measured as ß-gal activity in *M*. *smegmatis* and it revealed that the addition of the stem-loop resulted in a three-fold increase in expression, from 22.7±2.2 to 72.6±0.7 Miller units/mg protein. As the promoter regions in the two constructs were identical this strongly suggests that the stem-loop can confer stability to other transcripts than DrrS, and hence be applied to the engineered expression of RNA and/or proteins in mycobacteria.

## Discussion

Within the last decade it has become increasingly evident that post-transcriptional regulation of gene expression, including sRNA expression and sRNA/mRNA stability, contributes significantly to pathogen adaptation and survival. Nevertheless, knowledge about *M*. *tuberculosis* RNA biology is limited, and in order to fully understand the potential of this pathogen’s ability to adjust to the host environment we need to study these molecular mechanisms involved in regulating gene expression. To shed more light on these processes we characterised the expression, maturation, stability and turnover of DrrS, a DosR regulated sRNA from *M*. *tuberculosis*. We found that in *M*. *bovis* BCG, the activity of the main DrrS core promoter was significantly activated between exponential and stationary phase, suggesting that the promoter may be recognised by an alternative sigma factor, either in addition to, or instead of SigA, which is mostly log-phase associated [59]. However, scrutiny of the DrrS core promoter region did not reveal any obvious motifs for alternative sigma factors. Finally, we found that in *M*. *smegmatis* the DrrS core promoter was significantly activated (13–29 fold) in the presence of DosR_tb_, while the activity of the extended promoter region, which includes a known DBS, was only activated 5–8 fold and overall showed a decrease in promoter activity relative to the core promoter (Table 3). As this pattern was similar in two different strain backgrounds, i.e. mc^2^155 and Δ*dosR*_*sm*_ we believe that the results are authentic, but based on our current results we cannot determine if this is due to a novel molecular mechanism of DosR or if there are other factors involved. The weaker activation by DosR_tb_ in the presence of DosR_sm_ may be due to competition between the two factors, which have been shown to functionally overlap [60], although we would expect the level of DosR_tb_ to be significantly higher than DosR_sm_ due to the expression from a strong, exponential-phase promoter of the former. There are no reports on sigma factor expression being induced by DosR and hence we assume that activation of the DrrS core promoter by DosR is via a hitherto unknown molecular mechanism that does not require DBS. Some regulators stimulate transcription without DNA-binding but rather by directly interacting with RNA polymerase [61], although in our case it remains unclear how the promoter specificity would be achieved. It is possible that only promoters with -10 regions that diverge from the SigA consensus in a specific fashion, either by sequence or by spacing to the TSS would be recognised. The increased expression from the empty vector + *dosR*_*tb*_ suggests that the *dosR*_*tb*_ insert may contain a cryptic antisense promoter that drives low level expression of the *lacZ* reporter. However, the activity of the core promoter is still more than ten-fold higher than the empty vector, and hence the presence of such a cryptic promoter does not explain the dramatic activity of the core promoter in the presence of DosR_tb._

Overall our results suggest that DosR_tb_ activates the DrrS core promoter significantly, while the upstream region with the known DBS and the hypothetical DBS appears to reduce this expression under the conditions tested here. The activation of the core promoter by DosR may also explain why we observed an increase in DrrS core promoter activity in stationary phase in *M*. *bovis* BCG.

We have shown that DrrS is generated by rapid 3’ processing of a much longer transcript, DrrS+, which has no obvious canonical intrinsic terminator structure within the first 350 nucleotides. The absence of such a feature combined with the length of DrrS+ and the presence of multiple 3’ termini within DrrS+ raises the possibility that termination of DrrS transcription is Rho-dependent. The mature DrrS (108) is highly structured, while the 3’ domain of DrrS+ (from G165 to U297) is much less so, a feature known to facilitate the action of Rho [62]. The heterogeneous 3’ tails generated by Rho-dependent termination would explain the multiple signals observed by Northern blotting as well as a requirement for the observed 3’ processing at U108/C109 in order to obtain a well-defined 3’ terminus in the mature DrrS. Although there are no obvious Rho binding sites, i.e. low G, high C content [63], within the first 297 nucleotide DrrS+, we find a high C:G ratio in the 26 nucleotides immediately downstream. It therefore remains a possibility that Rho dependent termination is initiated in this region, while rapid processing precludes the detection of these transcripts.

Most known sRNAs are not 3’ processed as the poly-U tail that makes up part of the intrinsic terminator is crucial for binding of and regulation by the RNA chaperone, Hfq [64]. However, there is no Hfq homologue in *M*. *tuberculosis*; therefore, it is possible that such 3’ processing may be more widespread in this, and other species without Hfq. We have previously found several *M*. *tuberculosis* sRNAs that lack canonical intrinsic terminators at their mapped 3’ ends [14, 65] and unpublished, suggesting that these may also be subject to 3’ processing. Finally, it has been shown that transcription termination of a number of *E*. *coli* sRNAs is likely to involve Rho [66]. Many sRNAs were originally identified by predictive algorithms that used intrinsic terminators as one of the search parameters e.g. [67]; others were identified by their association with Hfq [68], which implies the presence of the poly-U tail of an intrinsic terminator. It is therefore probable that the use of more unbiased approaches such as RNA-seq and global TSS mapping [69], will lead to the identification of more sRNAs that lack intrinsic terminators.

**Fig 11:**
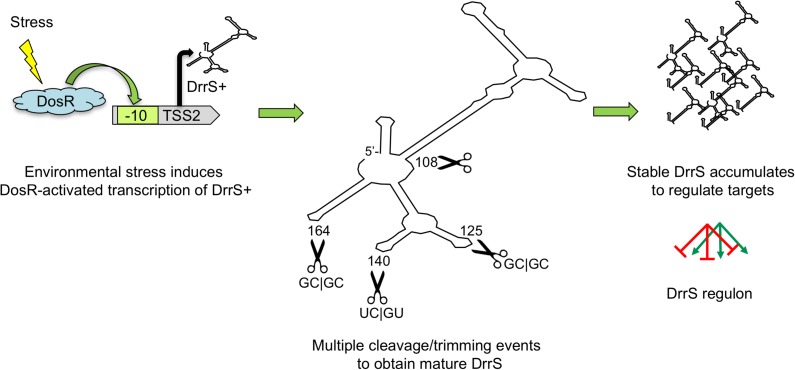
Cartoon showing the likely order of events in DrrS expression and function. Host-associated stress such as hypoxia or NO stress induces DosR expression and activation, which in turn leads to increased expression of DrrS+. This transcript is rapidly processed in multiple places to obtain the highly stable DrrS, which is able to regulate target genes for extended periods of time.

Although we assume that U108/C109 of the mature DrrS is generated by processing, we do not know which nuclease is involved, but the unpaired A110 followed by U111 could be susceptible to the action of both RNase E, RNase J. Similarly, we find that the high frequency of U residues in the 5’ processed DrrS variants suggests very strongly that either RNase E or RNase J may be involved. It is difficult to say if the 3’ ends beyond C122 have been generated by transcription termination, cleavage or trimming, respectively, but in this case the GC-rich nature of the majority of the mapped termini argues against the action of both RNase J and RNase E. Finally, although we have found very little evidence for the expression/existence of Rv1734c mRNA, it remains a possibility that the 3’ domain of DrrS+ and the Rv1734c mRNA form a perfect duplex that can be processed by RNase III. Future work may reveal how the primary DrrS+ and the mature DrrS regulate expression of target mRNAs. Our suggested model for the series of events has been shown in Fig 11.

DrrS accumulates to high levels in stationary phase, and this accumulation is due to a combination of the increased activity of the core promoter, and, more importantly, a very long half-life of DrrS, which can be mirrored in *M*. *smegmatis* using recombinant DrrS. We have shown that the 5’ stem-loop of DrrS contributes significantly to this stability and that the stability can be perturbed by the addition of two, or more nucleotides 5’ of the native TSS. As our 5’ RACE ruled out the removal of 5’ nucleotides by e.g. RNase J, our data strongly suggest that the 5’ phosphorylation state of the recombinant DrrS variants was modified by an RppH homologue, of which there is more than one candidate in *M*. *smegmatis*. Using RLM-RACE on *M*. *tuberculosis* sRNAs we have previously found that TAP dependent and TAP independent 5’ ends were identical, suggesting that tri- to mono-phosphate conversion by an RppH homologue also takes place in *M*. *tuberculosis* [14]. At this stage we cannot pinpoint the homologue responsible for the RppH activity in *M*. *smegmatis* nor in *M*. *tuberculosis*.

In this study we limited our DrrS 5’ additions to adenine residues to maintain the same initiating nucleotide in all the DrrS variants. Nevertheless, we still observe a significant reduction in RNA stability in A_3_DrrS and A_4_DrrS, which means that a guanine residue in the second position is not essential for de-phosphorylation, as it has been shown to be in *B*. *subtilis* [70, 71]. This could mean that the mycobacterial enzyme is more similar to the *E*. *coli* RppH, which does not display this preference [58], and our alignments did indeed indicate that the identified candidates showed more homology to *E*. *coli* than *B*. *subtilis* RppH. Alternatively, it may be that there is a level of redundancy between multiple enzymes in mycobacteria, similar to what has been reported for *B*. *subtilis* [56].

A fundamental question is how the pattern of DrrS expression and turnover may promote/facilitate *M*. *tuberculosis* growth and/or pathogenesis. During exponential growth, the expression of DrrS is on average < one copy per 10 cells, meaning its expression varies from one cell to the next, i.e. stochastic variation. The accumulation of DrrS is extremely slow, unlike many stress-induced sRNAs from Salmonella and *E*. *coli*, but the turnover of DrrS is also very slow, making the kinetics of this sRNA very unusual. As the cells enter stationary phase DrrS levels increase > 100-fold over the course of one week [13], which we have now shown is to a large extent due to its unrivalled stability. Although significant, this number also reflects that it takes at least 24 hours for DrrS levels to double. Moreover, it is highly likely that DrrS stability is further increased upon host-induced stress conditions such as hypoxia, which has been shown to stabilise *M*. *tuberculosis* transcripts on a global scale [52]. We did not observe a significant difference in DrrS stability between wildtype H37Rv and Δ*dosR*, thereby further supporting the notion that DrrS expression is DosR dependent. As DrrS turnover is very slow, it is tempting to speculate that DrrS (and possibly other, very stable *M*. *tuberculosis* RNAs), remains in the cell in times of non-replicating persistence when the rate of *de novo* RNA synthesis is low. DrrS would be able to post-transcriptionally modulate gene expression while those transcripts that depend on continuous RNA synthesis would disappear; similarly, one could also imagine that DrrS provides a long-term regulatory buffer that modulates translation of stochastically expressed transcripts during times of low metabolic activity or as the cells emerge from a dormant state. As *M*. *tuberculosis* is likely to be in a state of low metabolic activity most of the time and as DrrS remains in the cells for a considerable time after growth has re-initiated, it is conceivable that a minimum level of DrrS is present most of the time. Finally, as DrrS remains in the cells for prolonged periods after the addition of rifampicin, which is one of the first line drugs against TB, one could easily envision that DrrS remains, and plays a significant role within intracellular *M*. *tuberculosis* of patients undergoing chemotherapy with rifampicin. It is therefore important to investigate further what relationship there may be between drugs, DrrS accumulation and gene regulation.

In more general terms we have shown that a 5’ stem-loop results in a highly stable transcript similarly, but not identical to what has been shown in *E*. *coli* and in *B*. *subtilis*. In the case of *M*. *tuberculosis* DrrS we find that the stem-loop contributes to a half-life that is in excess of 6 hours. Our results also show how the accumulation and abundance of DrrS is highly dependent on this stability. Thus, based on Northern blotting, we estimate that the level of wildtype DrrS is at least 100-fold higher than the level of A_4_DrrS when expressed from the same, strong promoter (Fig 9B). Moreover, we have shown that the basic stem-loop could be added to another (translated) transcript to enhance its expression. This insight can be applied to modify the engineered expression of *M*. *tuberculosis* genes in combination with other means of regulation. For example, the expression from inducible promoters can be increased without increasing promoter activity *per se*, as this may lead to more leaky expression. Similarly, a long-lived mRNA may be desirable for protein expression in recombinant live vaccines. Conversely, a very unstable mRNA may be desirable for other purposes. Future investigations based on global TSS mapping and RNA structure predictions, may reveal if stable *M*. *tuberculosis* transcripts contain a similar 5’ stem-loop.

This investigation provides a comprehensive insight into several general aspects of *M*. *tuberculosis* RNA biology. We have shown that RNA stability/turnover can be at least as important as promoter activity in determining the final outcome of RNA levels, as seen by the decrease in total transcript levels between DrrS and A_3_DrrS and A_4_DrrS. We have shown that a simple stem-loop can enhance the expression of a heterologous gene. We have also shown that *M*. *tuberculosis* sRNAs can be generated by 3’ processing. These characteristics are likely to apply to many other sRNAs and mRNAs, and this study will hopefully provide a stepping stone for further studies of *M*. *tuberculosis* RNA as well as the proteins that interact with it.

## Materials and methods

### Bacterial strains and growth conditions

*Escherichia coli* DH5α was grown in LB liquid culture or agar (1.5%) supplemented with 50 μg/ml kanamycin or 250 μg/ml hygromycin B as required.

*Mycobacterium smegmatis* mc^2^155 was grown on LB agar supplemented with 25 μg/ml kanamycin or 50 μg/ml hygromycin B, and in liquid Middlebrook 7H9 medium supplemented with 10% Albumin, 0.2% glycerol, 0.02% Tween 80 and 20 μg/ml kanamycin or 50 μg/ml hygromycin B as required.

*Mycobacterium tuberculosis* H37Rv and *Mycobacterium bovis* BCG were grown on Middlebrook 7H11 agar supplemented with 10% OADC, 0.4% glycerol and 20 μg/ml kanamycin or 50 μg/ml hygromycin B as required and in liquid Middlebrook 7H9 medium supplemented with 10% ADC, 0.02% glycerol and 0.02% Tween 80 in roller bottles (Cell Master, Griener Bio-One) or PETG flasks (Nalgene, Thermo Scientific), respectively. For all three mycobacterial species exponential phase cultures were harvested at 0.5 < OD_600_ < 0.8. Stationary phase cultures for *M*. *tuberculosis* and *M*. *bovis* BCG were harvested at least 1 week after OD_600_ = 1.0. Time course experiments cultures were harvested as indicated

Nitric oxide stress was induced by adding DETA-NO (Sigma-Aldrich) directly to cultures at a final concentration of 0.1 mM or 1.0 mM, respectively for 2 hours before RNA was isolated. To inhibit transcription initiation rifampicin was added to cultures at a final concentration of 200 μg/ml.

### Oligonucleotides

Oligonucleotides used during this study are listed in S3 Table

### Plasmid construction

Plasmids used in this study are listed in S4 Table

pKA425 was derived from pEJ425 [72], by site directed mutagenesis of the sequence surrounding the *lacZ* start codon changing AAATGG to CCATGG, to include an NcoI site for translational fusions. pIRaTE (Integrating Regulator and Target Expression vector) was created by inserting a gBlocks Gene fragment (IDT) flanked with AgeI and NcoI sites into the XbaI and NcoI sites of pKA425. The pIRaTE gBlock fragment contains two divergently transcribed regions for target and regulator insertion, respectively. The regulator side contains a synthetic terminator SynB [47] and fragments were cloned (via the SpeI site) such that the native transcription start site was upheld. In addition, promoter inserts were exchanged in order to modify expression levels of both regulator and target.

The *M*. *tuberculosis* DrrS was expressed from the *M*. *smegmatis* ribosomal *rrnB* promoter spanning -80 to -8 [48]. The DrrS overexpression constructs for wild type, 5’ and 3’ variants were cloned into pIRaTE as SpeI-XhoI fragments. The wild type DrrS construct was made by PCR amplification (using DrrS.wtf with DrrS.wtr) from H37Rv genomic DNA. DrrS wild type plasmid was subsequently used as a template for both 5’ and 3’ variant construction. The 5’ and 3’ variants were created with oligos listed in S3 Table. After cloning in *E*. *coli* and sequencing, the constructs were electroporated into *M*. *smegmatis* mc^2^155.

DrrS promoter and stem loop fusions to *lacZ* were created by either oligonucleotide annealing or PCR amplification using the primer pairs specified in S1 Table. Each fragment outlined above was cloned into pEJ414 [73] as XbaI-HindIII fragments. The *M*. *tuberculosis dosR* coding sequence was expressed from the *M*. *chelonae* PCL1 core promoter [74], followed by the SynB synthetic terminator [47]. This was created as a gBlock fragment (IDT technologies) flanked with XbaI sites and cloned upstream of the *lacZ* reporter so that transcription was divergent from this.

After verification of the sequences, the DrrS promoter reporter constructs were electroporated into either *M*. *smegmatis* mc^2^155 or *ΔdosR* or *M*. *bovis* BCG.

### RNA isolation

Total RNA extraction was performed as previously described [13, 14]. Briefly, ice was added directly to the culture, which was centrifuged at 5000 rpm for 10 minutes at 2°C and total RNA was extracted using the FastRNA Pro Blue Kit (MP Bio) according to the manufacturer’s instructions. RNA concentration and quality was determined using a Nanodrop 2000.

### cDNA synthesis, 3’ and 5’ RACE (Rapid amplification of cDNA ends)

DNA was removed from total RNA by treating 10 μg of total RNA with 2 U Turbo DNase I (Ambion) for 30 minutes at 37°C, then adding an additional 2 U DNase I for a further 30 minutes followed by extraction with acidic phenol-chloroform and ethanol precipitation. cDNA was synthesised using Superscript III (Invitrogen), with oligonucleotides described below, largely according to manufacturer’s instruction except for an additional extension step for 30 minutes at 55°C.

3’ RACE was performed by poly(A) tailing total RNA using *E*. *coli* poly-A-polymerase (Ambion), following manufacturer’s instructions. cDNA was synthesised using the oligo(d)T primer, and PCRs were done using Red-Taq DNA polymerase (Sigma) with primers shown in S1 Table.

For 5’ RACE, in order to enable linker ligation, total RNA was treated with RNA 5’ polyphosphatase (Epicentre/Cambio) in line with manufacturer’s instructions. An RNA linker was ligated to the RNA using T4 RNA ligase (NEB) at 17°C overnight. cDNA synthesis was performed using GR5’ as described, followed by PCR amplification using GR5’ with DrrS.5’RACEr (S1 Table) and Red-Taq (Sigma). PCR reactions were separated on a 3% agarose gel (QA high resolution, MP Bio), and bands of interested were excised and purified with Qiagen Gel Extraction kit before cloning.

For 3’ RACE, PCR products were cloned into TOPO TA sequencing vector pCRII-TOPO (Invitrogen) and sequenced by Source Bioscience.

### Northern blotting

Total RNA was separated on 8%, 10% or 15% denaturing acrylamide gels and transferred onto Brightstar-Plus nylon membrane (Ambion) by electroblotting. RNA was UV cross-linked to the membrane then stained with 0.3 M sodium acetate/0.03% methylene blue to verify transfer. ^32^P-UTP labelled riboprobes were synthesised using the mirVana Probe construction Kit (Ambion) and ^32^P-UTP (800 mCi/mmol, PerkinElmer) with oligos listed in S1 Table, and hybridised to the membranes overnight in UltraHyb (Invitrogen). RNA sizes were compared to 100–500 nucleotide Century marker (Ambion) or 50–500 nucleotide low range ssRNA ladder (NEB).

### ß-galactosidase assay

ß-galactosidase assays were carried out as described previously [48]. Briefly, cultures were cooled on ice prior to centrifugation at 2°C for 10 minutes, pellets were washed three times with ice cold Z-buffer (40 mM NaH_2_PO_4_.H_2_O, 60 mM Na_2_HPO_4_.7H_2_O, 1 mM MgSO_4_.7H_2_O, 10 mM KCl) before disrupting with 150–212 μM acid washed glass beads (Sigma-Aldrich) in a FastPrep instrument (MP Bio). Disrupted cells were centrifuged for 10 minutes at 13 000rpm at 2°C and the supernatant was retained; an aliquot of each extract was used to determine the total protein concentration using a BCA protein assay kit (Pierce), ß-mercaptoethanol was added to the remainder at a final concentration of 38 mM. ß-galactosidase activity was determined as described [75], normalized to total protein concentration and expressed as units/(mg protein). Indicated values represent at least three biological replicates.

## Supporting information

S1 Fig Extended time course of DrrS accumulation until three weeks past log-phase. Membrane shows DrrS and 5S loading control at the same time verifying that DrrS is ~110 nucleotides in size.
(TIF)

S2 Fig Turnover of DrrS in stationary phase in H37Rv and Δ*dosR*. Rifampicin (200 μg/ml) was added to stationary phase cultures of *M*. *tuberculosis* and RNA was harvested at the indicated time points and analysed by Northern blotting.
(TIF)

S3 Fig RppH candidates in *M*. *smegmatis*. Phylogenetic tree of *E*. *coli* RppH and *M*. *smegmatis* RppH candidates (i.e. NUDIX hydrolases) performed with EBI Clustal Omega (http://www.ebi.ac.uk/Tools/msa/clustalo/).
(TIFF)

S1 Table 3’ RACE of DrrS variants expressed in *M*. *smegmatis*. (DOCX)

S2 Table Extended 3’ RACE of wildtype DrrS from *M*. *tuberculosis*. (DOCX)

S3 Table DNA oligos used in this study. (DOCX)

S4 Table Plasmids used in this study. (DOCX)
