# Supplementary figures and images for: Expression, maturation and turnover of DrrS, an unusually stable, DosR regulated small RNA in Mycobacterium tuberculosis
Source: PLoS One. 2017 Mar 21;12(3):e0174079. doi: 10.1371/journal.pone.0174079 (PMC5360333; doi:10.1371/journal.pone.0174079)

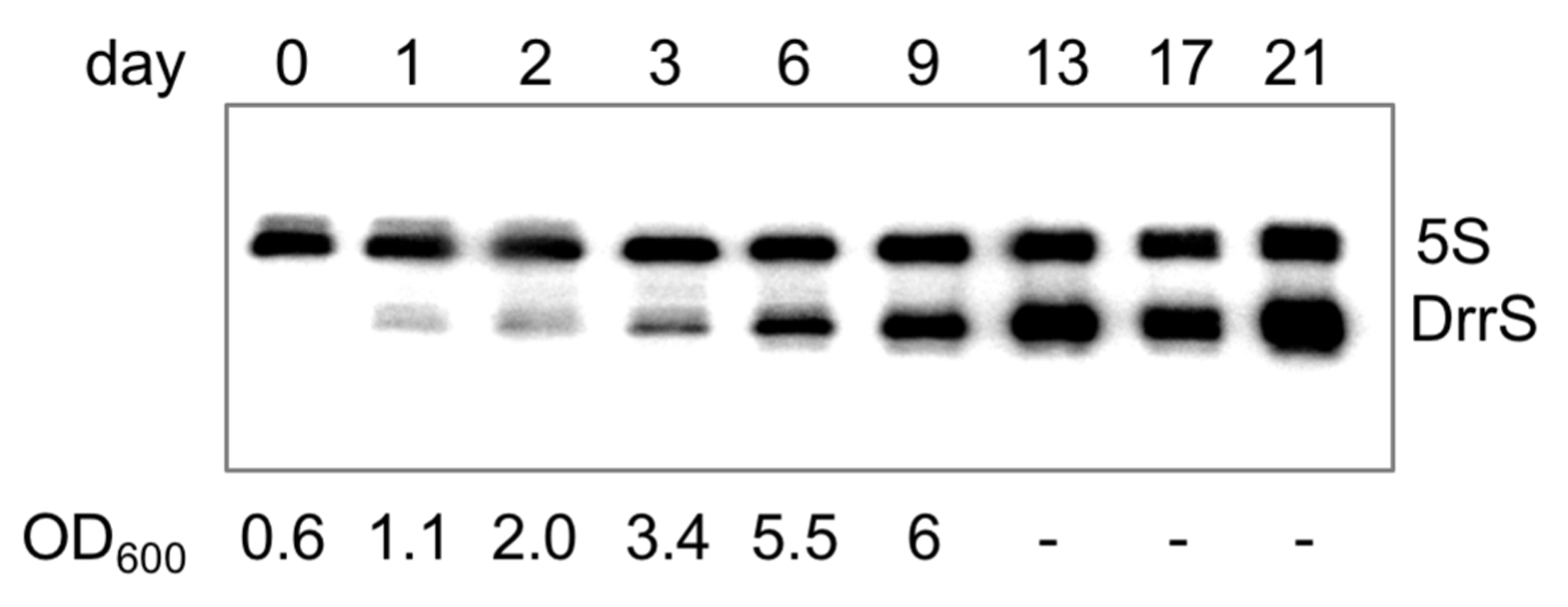

Supplement: S1 Fig — Membrane shows DrrS and 5S loading control at the same time verifying that DrrS is ~110 nucleotides in size. (TIF) [file pone.0174079.s001.tif]

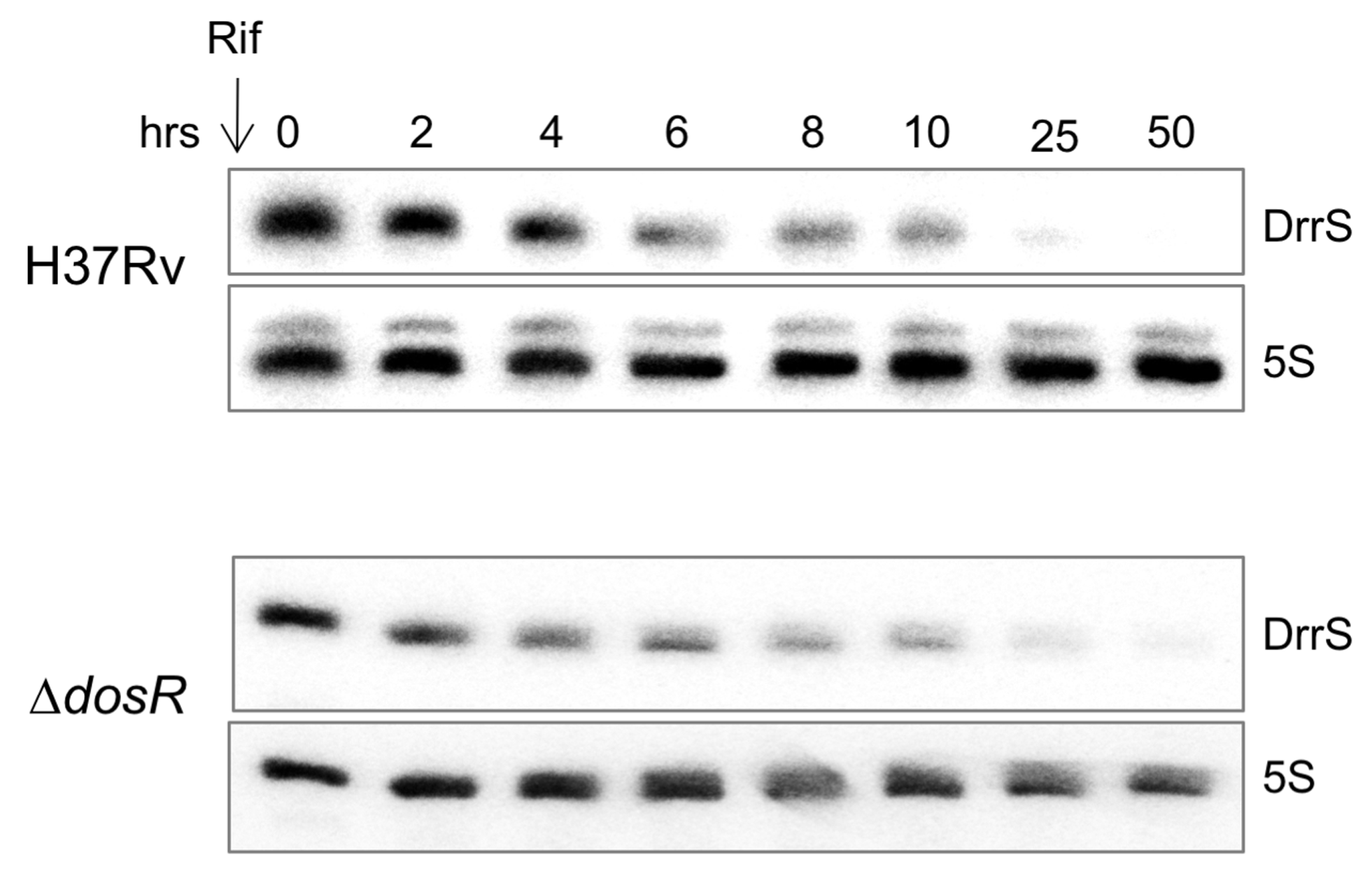

Supplement: S2 Fig — Rifampicin (200 μg/ml) was added to stationary phase cultures of M. tuberculosis and RNA was harvested at the indicated time points and analysed by Northern blotting. (TIF) [file pone.0174079.s002.tif]

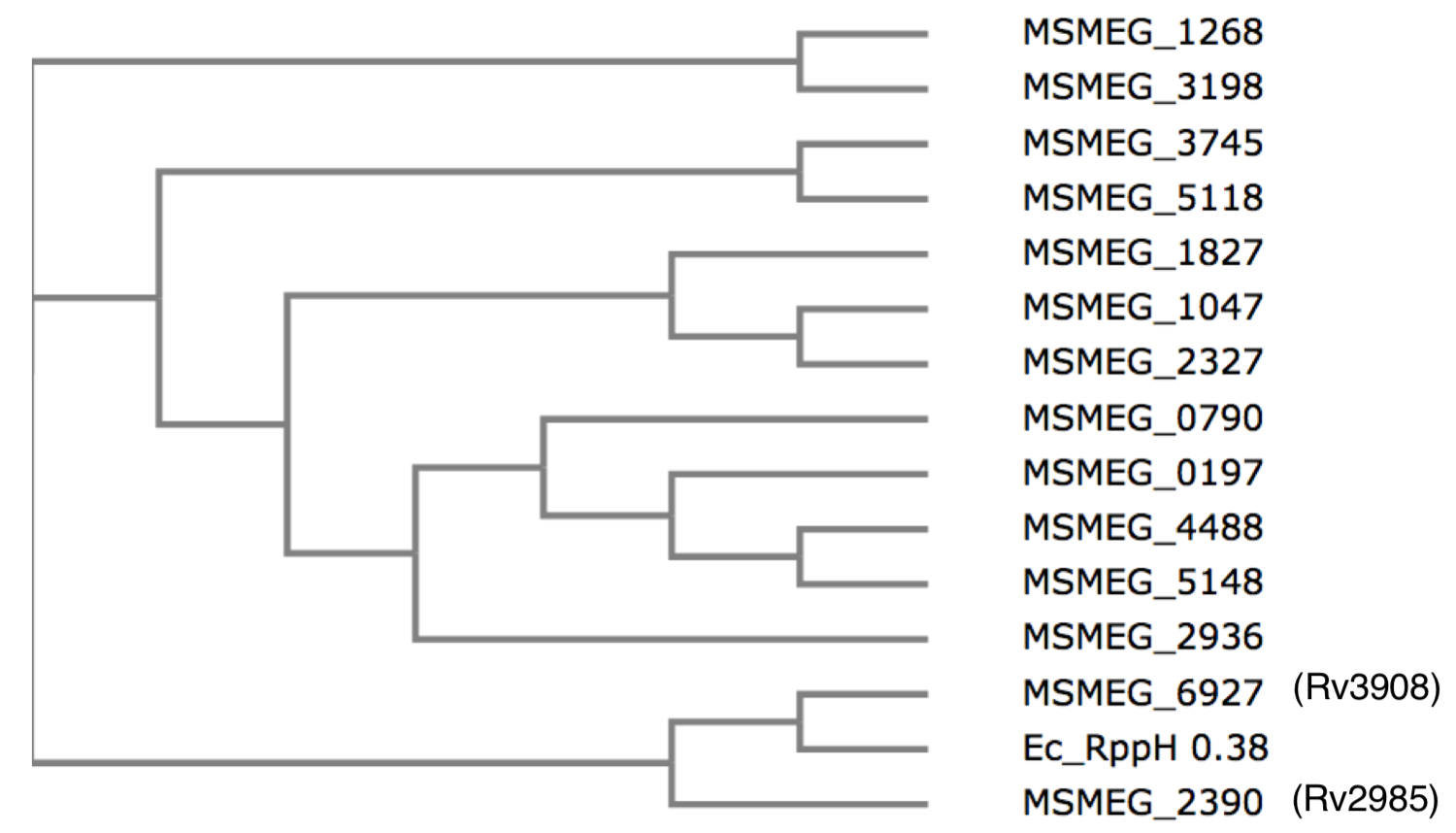

Supplement: S3 Fig — Phylogenetic tree of E. coli RppH and M. smegmatis RppH candidates (i.e. NUDIX hydrolases) performed with EBI Clustal Omega (http://www.ebi.ac.uk/Tools/msa/clustalo/). (TIFF) [file pone.0174079.s003.tiff]
