# Supplementary material for: Expression, maturation and turnover of DrrS, an unusually stable, DosR regulated small RNA in Mycobacterium tuberculosis
Source: PLoS One. 2017 Mar 21;12(3):e0174079. doi: 10.1371/journal.pone.0174079 (PMC5360333; doi:10.1371/journal.pone.0174079)
Supplement: S1 Table — (DOCX) [file pone.0174079.s004.docx]

Table S1

| **DNA sequence** | **DrrS position** | | | | **108** | **109** | **110** | **111** | **112** | **113** | **114** | **115** | **Total** |
| --- | --- | --- | --- | --- | --- | --- | --- | --- | --- | --- | --- | --- | --- |
| Willdtype 3’ end  (2 repeats)  **aTCCTCaTCCTC** | Sequence | U | C | C | **U** | C | A | U | C | C | U | C |  |
|  | % |  |  |  | **85** | 9 | - | 3 | 3 |  |  |  |  |
|  | n of clones |  |  |  | 29 | 3 |  | 1 | 1 |  |  |  | 34 |
| Extended 3’ end  (3 repeats)  **aTCCTCaTCCTCaTCCT** | Sequence | U | C | C | U | **C** | A | U | C | C | U | C |  |
|  | % |  |  | 5 | 21 | **42** | - | 11 | 5 | 5 | 11 |  |  |
|  | n of clones |  |  | **1** | 4 | 8 |  | 2 | 1 | 1 | 2 |  | 19 |
| Cropped 3’ end  (1 repeat)  **aTCCT** | Sequence | U | C | C | **U** | C | U | C | G | A | G | A |  |
|  | % |  |  |  | **33** | 6 |  | 28 | 28 | - | 6 | - |  |
|  | n of clones |  |  |  | 6 | 1 |  | 5 | 5 |  | 1 |  | 18 |

3’ RACE results of DrrS variants expressed in *M. smegmatis*. A-residues were not assigned as the RACE was done by adding a poly-A tail.
