# Supplementary material for: Expression, maturation and turnover of DrrS, an unusually stable, DosR regulated small RNA in Mycobacterium tuberculosis
Source: PLoS One. 2017 Mar 21;12(3):e0174079. doi: 10.1371/journal.pone.0174079 (PMC5360333; doi:10.1371/journal.pone.0174079)
Supplement: S2 Table — (DOCX) [file pone.0174079.s005.docx]

**Table S2: extended 3’ RACE of DrrS**

| **Clone No.** | **Position from TSS** | **No. of clones/total** | **Two nt*** | **Four nt*** |
| --- | --- | --- | --- | --- |
| **Extension 1 (CTTCGGGCAGATCCTCATCCTGTTAC)** | | | |  |
| 1 | 122 | 1/15 | **C\|G** | **GC\|GG** |
| 2 | 123 | 1/15 | g\|g | cg\|gc |
| 3 | 125 | 3/15 | **C\|G** | **GC\|GC** |
| 4 |  |  |  |  |
| 5 |  |  |  |  |
| 6 | 135 | 2/15 | c\|a/a\|g | uc\|ag / ca\|gc |
| 7 |  |  |  |  |
| 8 | 162 | 1/15 | **C\|G** | **GC\|GC** |
| 9 | 164 | 2/15 | **C\|G** | **GC\|GC** |
| 10 |  |  |  |  |
| 11 | 198 | 1/15 | g\|g | cg\|gg |
| 12 | 204 | 1/15 | **C\|G** | **GC\|GU** |
| 13 | 215 | 1/15 | **C\|G** | **AC\|GG** |
| 14 | 227 | 1/15 | **C\|G** | **AC\|GU** |
| 15 | 228 | 1/15 | g\|u | cg\|ug |
| **Extension 2 (GTTACTGCGGCGCACCGCGTC)** | | | |  |
| 16 | 138 | 1/21 | c\|u | gc\|uc |
| 17 | 140 | 3/21 | **C\|G** | **UC\|GU** |
| 18 |  |  |  |  |
| 19 |  |  |  |  |
| 20 | 142 | 1/21 | u\|u | gu\|ug |
| 21 | 146 | 1/21 | u\|c | au\|cg |
| 22 | 148 | 1/21 | g\|a | cg\|ac |
| 23 | 162 | 2/21 | **C\|G** | **GC\|GC** |
| 24 |  |  |  |  |
| 25 | 164 | 4/21 | **C\|G** | **GC\|GC** |
| 26 |  |  |  |  |
| 27 |  |  |  |  |
| 28 |  |  |  |  |
| 29 | 212 | 1/21 | g\|c | ag\|ca |
| 30 | 228 | 1/21 | g\|u | cg\|ug |
| 31 | 258 | 1/21 | u\|u | gu\|ug |
| 32 | 259 | 1/21 | u\|g | uu\|gu |
| 33 | 261 | 1/21 | u\|g | gu\|gc |
| 34 | 271 | 1/21 | c\|c | gc\|cg |
| 35 | 273 | 1/21 | g\|a/a\|a | cg\|aa / ga\|aa |
| 36 | 297 | 1/21 | u\|g | gu\|gg |

* Two nt and four nt refer to the cleavage sites seen in a two-nucleotide and four-nuclotide context, respectively
