# Supplementary material for: Expression, maturation and turnover of DrrS, an unusually stable, DosR regulated small RNA in Mycobacterium tuberculosis
Source: PLoS One. 2017 Mar 21;12(3):e0174079. doi: 10.1371/journal.pone.0174079 (PMC5360333; doi:10.1371/journal.pone.0174079)
Supplement: S3 Table — (DOCX) [file pone.0174079.s006.docx]

Table S3

| **Oligonucleotide name** | **Sequence** |
| --- | --- |
| **Riboprobes** | |
| 5S_RP | GTCCCATTCCGAACCCGGAAGCTAAGCCTGCCAGCGCCTGTCTC |
| DrrS_RP | GATCTGCCCGAAGTGCTGGGCGATTGAGCGGGTATGTACACCTGTCTC |
| DrrS_RPExt | GACCTACCGTCCCAAGACGGGGCTACCGCCTTCGGGCAGACCTGTCTC |
| MTS2823RP | GCACCCACGCGGAGTCATAGCCACGATAACGGCAGAAGCCTGTCTC |
| **DrrS promoter fusions in pEJ414** | |
| DrrS.coreF | CTAGATCCCGCCTGCTGTGTTTGGTGGCAGTATTGGTGATA |
| DrrS.coreR | AGCTTATCACCAATACTGCCACCAAACACAGCAGGCGGGAT |
| DrrS.coreStemloopf | CTAGATCCCGCCTGCTGTGTTTGGTGGCAGTATTGGTGATACCGGGGAAACCCGGTA |
| DrrS.coreStemloopr | AGCTTACCGGGTTTCCCCGGTATCACCAATACTGCCACCAAACACAGCAGGCGGGAT |
| DrrS.DosR2f | CTAGACAGGCGAAGTTTTATTGTCGGATAAGGGACTTTCGCCCCTTCCCGCCTGCTGTGTTTGGTGGCAGTATTGGTGATA |
| DrrS.DosR2r | AGCTTATCACCAATACTGCCACCAAACACAGCAGGCGGGAAGGGGCGAAAGTCCCTTATCCGACAATAAAACTTCGCCTGT |
| DrrS.DosR2Stemloopf | CTAGACAGGCGAAGTTTTATTGTCGGATAAGGGACTTTCGCCCCTTCCCGCCTGCTGTGTTTGGTGGCAGTATTGGTGATACCGGGGAAACCCGGTA |
| DrrS.DosR2Stemloopr | AGCTTACCGGGTTTCCCCGGTATCACCAATACTGCCACCAAACACAGCAGGCGGGAAGGGGCGAAAGTCCCTTATCCGACAATAAAACTTCGCCTGT |
| **DrrS wt, 3’ variants and 5’extended in pIRaTE** | |
| DrrS.wtf | GCGCTCTCGAGAGGATGAGGAT |
| DrrS.wtr | TAACTTACTAGTACCGGGGAAACCC |
| DrrS.extended3’f | TATACTCGAGAGGATGAGGATGAGGATCT |
| DrrS.cropped3’f | TATACTCGAGAGGATCTGCCCGAAG |
| DrrS.3’r | TAATCCATGGTAGCTTCCTTAGCTCCTG |
| DrrS.5’A_1_r | TAACTTACTAGTAACCGGGGAAACCC |
| DrrS.5’A_2_r | TAACTTACTAGTAAACCGGGGAAACCC |
| DrrS.5’A_3_r | TAACTTACTAGTAAAACCGGGGAAACCC |
| DrrS.5’A_4_r | TAACTTACTAGTAAAAACCGGGGAAACCC |
| **3’ and 5’ RACE oligonucleotides** | |
| DrrS.3’RACEf | GATCTGCCCGAAGTGCTGGGCGAT |
| DrrS.Ext1RACEf | CTTCGGGCAGATCCTCATCCTGTTAC |
| DrrS.Ext2RACEf | GTTACTGCGGCGCACCGCGTC |
| Oligo(d)T | GCTGTCAACGATACGCTACGTAACGGCATGACAGTGT_24_ |
| GeneRacer3’f | GCTGTCAACGATACGCTACGTAACG |
| GR5’ | CGACTGGAGCACGAGGACACTGA |
| RNA linker | CGACUGGAGCACGAGGACACUGACAUGGACUGAAGGAGUAGAAA |
| DrrS.5’RACEr | AGGATCTGCCCGAAGGCGGTAGC |
| Mint | ACGAGGGGCATTCACACCAGATTG |
| 518R | TTTCCCAGTCACGACGTTGTAAAA |
