# Supplementary material for: Expression, maturation and turnover of DrrS, an unusually stable, DosR regulated small RNA in Mycobacterium tuberculosis
Source: PLoS One. 2017 Mar 21;12(3):e0174079. doi: 10.1371/journal.pone.0174079 (PMC5360333; doi:10.1371/journal.pone.0174079)
Supplement: S4 Table — (DOCX) [file pone.0174079.s007.docx]

| **Table S4** | | | | |
| --- | --- | --- | --- | --- |
| **Plasmid ID** | **Plasmid name** | **Construction** | **Origin, Marker** | **Reference** |
| pEJ414 |  | Integrating promoterless *lacZ* reporter vector | Kan | [73] |
| pAM100 | DrrS.core | Transcriptional fusion of DrrS core promoter region (-35 to +1) to *lacZ*; insert amplified with DrrS.coref/DrrS.corer | pEJ414, KanR | This study |
| pAM101 | DrrS.DBS2 | Transcriptional fusion of DrrS core promoter region with 1DBS and 2^nd^ putative DosR binding site (-75 to +1) to *lacZ*; insert amplified with DrrS.DosR2f/ DrrS.corer | pEJ414, KanR | This study |
| pAM102 | DrrS.coreStemloop | Transcriptional fusion of DrrS core promoter region with the addition of DrrS 5’ stem loop (ACCGGGGAAACCCGGT) to *lacZ*; insert amplified with DrrS.coreStemloopf/DrrS.coreStemloopr | pAM100, Kan | This study |
| pAM103 | DrrS.DBS2Stemloop | Transcriptional fusion of DrrS core promoter region with 1DBS and 2^nd^ putative DosR binding site with the addition of DrrS 5’ stem loop (ACCGGGGAAACCCGGT) to *lacZ*; insert amplified with DrrS.DosR2Stemloopf/ DrrS.DosR2Stemloopr | pAM101, Kan | This study |
| pEJ425 |  | Integrating promoterless *lacZ* reporter vector | pEJ414, HygR | [72] |
| pKA425 |  | Integrating promoterless *lacZ* reporter vector with NcoI site at lacZ ATG | pEJ425, HygR | This Study |
| pIRaTE |  | Integrating Regulator and Target Expression control plasmid, regulator overexpressed from *rrnB* (-80 to -8) promoter and terminated by SynB synthetic terminator | pKA425, HygR | This study |
| pIRaTE.001 | DrrSwt | Wild type DrrS (+1 to +114) overexpressed from *rrnB* promoter; insert amplified with DrrS.wtf/DrrS.wtr | pIRaTE, HygR | This study |
| pIRaTE.002 | DrrS.extended3’ | DrrS with an extended 3’end (+1 to +114 plus CATCCT), overexpressed from *rrnB* promoter; insert amplified with DrrS.extended3’f/DrrS.wtr | pIRaTE.001, HygR | This study |
| pIRaTE.003 | DrrS.cropped3’ | DrrS with a cropped 3’end (+1 to 109), overexpressed from *rrnB* promoter; insert amplified with DrrS.cropped3’f/DrrS.wtr | pIRaTE.001, HygR | This study |
| pIRaTE.004 | DrrS.5’A_1_ | DrrS (+1 to +114) extended at the 5’end with one adenine (A), overexpressed from *rrnB* promoter; insert amplified with DrrS.wtf/DrrS.5’A_1_r | pIRaTE, HygR | This study |
| pIRaTE.005 | DrrS.5’A_2_ | DrrS (+1 to +114) extended at the 5’end with two adenines (AA), overexpressed from *rrnB* promoter; insert amplified with DrrS.wtf/DrrS.5’A_2_r | pIRaTE, HygR | This study |
| pIRaTE.006 | DrrS.5’A_3_ | DrrS (+1 to +114) extended at the 5’end with three adenines (AAA), overexpressed from *rrnB* promoter; insert amplified with DrrS.wtf/DrrS.5’A_3_r | pIRaTE, HygR | This study |
| pIRaTE.007 | DrrS.5’A_4_ | DrrS (+1 to +114) extended at the 5’end with four adenines (AAAA), overexpressed from *rrnB* promoter; insert amplified with DrrS.wtf/DrrS.5’A_4_r | pIRaTE, HygR | This study |
